# Hepcidin-guided screen-and-treat interventions against iron-deficiency anaemia in pregnancy: a randomised controlled trial in The Gambia

**DOI:** 10.1016/S2214-109X(19)30393-6

**Published:** 2019-10-10

**Authors:** Amat Bah, Abdul Khalie Muhammad, Rita Wegmuller, Hans Verhoef, Morgan M Goheen, Saikou Sanyang, Ebrima Danso, Ebrima A Sise, Sant-Rayn Pasricha, Andrew E Armitage, Hal Drakesmith, James H Cross, Sophie E Moore, Carla Cerami, Andrew M Prentice

**Affiliations:** a Medical Research Council (MRC) Unit The Gambia at London School of Hygiene & Tropical Medicine (LSHTM), Serrekunda, The Gambia; b Division of Human Nutrition and Health, Wageningen University, Wageningen, Netherlands; c Population Health and Immunity Division, The Walter and Eliza Hall Institute of Medical Research, and Department of Medical Biology, University of Melbourne, Melbourne, VIC, Australia; d MRC Human Immunology Unit, MRC Weatherall Institute of Molecular Medicine, University of Oxford, John Radcliffe Hospital, Oxford, UK; e LSHTM, London, UK; f GroundWork, Flaesch, Switzerland; g University of North Carolina at Chapel Hill School of Medicine, Department of Microbiology and Immunology, Chapel Hill, NC, USA; h Department of Women & Children's Health, King's College London, St Thomas' Hospital, London, UK

## Abstract

**Background:**

WHO recommends daily iron supplementation for pregnant women, but adherence is poor because of side-effects, effectiveness is low, and there are concerns about possible harm. The iron-regulatory hormone hepcidin can signal when an individual is ready-and-safe to receive iron. We tested whether a hepcidin-guided screen-and-treat approach to combat iron-deficiency anaemia could achieve equivalent efficacy to universal administration, but with lower exposure to iron.

**Methods:**

We did a three-arm, randomised, double-blind, non-inferiority trial in 19 rural communities in the Jarra West and Kiang East districts of The Gambia. Eligible participants were pregnant women aged 18–45 years at between 14 weeks and 22 weeks of gestation. We randomly allocated women to either WHO's recommended regimen (ie, a daily UN University, UNICEF, and WHO international multiple-micronutrient preparation [UNIMMAP] containing 60 mg iron), a 60 mg screen-and-treat approach (ie, daily UNIMMAP containing 60 mg iron for 7 days if weekly hepcidin was <2·5 μg/L or UNIMMAP without iron if hepcidin was ≥2·5 μg/L), or a 30 mg screen-and-treat approach (ie, daily UNIMMAP containing 30 mg iron for 7 days if weekly hepcidin was <2·5 μg/L or UNIMMAP without iron if hepcidin was ≥2·5 μg/L). We used a block design stratified by amount of haemoglobin at enrolment (above and below the median amount of haemoglobin on every enrolment day) and stage of gestation (14–18 weeks *vs* 19–22 weeks). Participants and investigators were unaware of the random allocation. The primary outcome was the amount of haemoglobin at day 84 and was measured as the difference in haemoglobin in each screen-and-treat group compared with WHO's recommended regimen; the non-inferiority margin was set at −5·0 g/L. The primary outcome was assessed in the per-protocol population, which comprised all women who completed the study. This trial is registered with the ISRCTN registry, number ISRCTN21955180.

**Findings:**

Between June 16, 2014, and March 3, 2016, 498 participants were randomised, of whom 167 were allocated to WHO's recommended regimen, 166 were allocated to the 60 mg per day screen-and-treat approach, and 165 were allocated to the 30 mg per day screen-and-treat approach. 78 participants were withdrawn or lost to follow-up during the study; thus, the per-protocol population comprised 140 women assigned to WHO's recommended regimen, 133 allocated to the 60 mg screen-and-treat approach, and 147 allocated to the 30 mg screen-and-treat approach. The screen-and-treat approaches did not exceed the non-inferiority margin. Compared with WHO's recommended regimen, the difference in the amount of haemoglobin at day 84 was −2·2 g/L (95% CI −4·6 to 0·1) with the 60 mg screen-and-treat approach and −2·7 g/L (–5·0 to −0·5) with the 30 mg screen-and-treat approach. Adherence, reported side-effects, and adverse events were similar between the three groups. The most frequent side-effect was stomachache, which was similar in the 60 mg screen-and-treat group (82 cases per 1906 person-weeks) and with WHO's recommended regimen (81 cases per 1974 person-weeks; effect 1·0, 95% CI 0·7 to 1·6); in the 30 mg screen-and-treat group the frequency of stomachache was slightly lower than with WHO's recommended regimen (58 cases per 2009 person-weeks; effect 0·7, 95% CI 0·5 to 1·1). No participants died during the study.

**Interpretation:**

The hepcidin-guided screen-and-treat approaches had no advantages over WHO's recommended regimen in terms of adherence, side-effects, or safety outcomes. Our results suggest that the current WHO policy for iron administration to pregnant women should remain unchanged while more effective approaches continue to be sought.

**Funding:**

Bill & Melinda Gates Foundation and the UK Medical Research Council.

## Introduction

Iron deficiency and associated anaemia is the most prevalent micronutrient deficiency worldwide, affecting an estimated 1·24 billion people.^1^ It is the leading cause of years lived with disability in most of sub-Saharan Africa and many parts of Asia.^1^ WHO recommends universal daily iron and folic acid supplementation in pregnancy,^2^ based on evidence from a Cochrane review that it provides maternal and neonatal health benefits^3^ such as prevention of maternal anaemia, puerperal sepsis, low birthweight, and preterm birth. The recommended daily dose is 30–60 mg elemental iron, with a preferred dose of 60 mg per day in countries where anaemia prevalence exceeds 40%.^2^ In low-income and middle-income countries (LMICs), iron and folic acid supplementation has great benefits for iron-deficient women^4^ and is increasingly being combined in multiple micronutrient formulations.^5^ However, even when supplements are made available, implementation of a daily supplementation policy in LMICs is highly variable and adherence to a daily regimen is poor,^4^, ^5^, ^6^, ^7^ attributable in large part to common gastrointestinal side-effects such as constipation, nausea, vomiting, black stools, and epigastric discomfort.^8^, ^9^ WHO subsequently recommended intermittent supplementation if daily iron caused side-effects.^10^, ^11^ Concerns have also been reported that iron supplementation can predispose to haemoconcentration^3^ and gestational diabetes.^12^ In low-income settings, an additional possibility is that iron supplementation might increase gastrointestinal and other infections,^4^ particularly malaria. Anaemia and low iron status are associated with protection against falciparum malaria in pregnant women,^13^, ^14^ and there are clear pathways by which iron administration abrogates this protection.^15^ Thus, lowering the dose of supplemental iron could be beneficial if it could be achieved without compromising efficacy.

**Research in context**

**Evidence before this study**

Based on regularly updated meta-analyses, WHO recommends that pregnant women should take supplements containing 30–60 mg elemental iron and 400 μg folic acid daily to prevent maternal anaemia, puerperal sepsis, low birthweight, and preterm birth. If daily supplementation is not acceptable because of side-effects, weekly supplementation with 120 mg iron and 2800 μg folic acid is a suitable alternative in settings where the prevalence of anaemia is less than 20%. Side-effects are frequently reported, however, and contribute to poor adherence. An additional concern is that iron might predispose to gestational diabetes, pre-eclampsia, and infections. We reasoned that a screen-and-treat approach to combat anaemia in pregnancy would be advantageous if it could achieve efficacy that was equivalent to or better than that achieved by WHO's recommendations, but at a lower overall dose of iron and with fewer side-effects.

**Added value of this study**

We did a double-blind randomised trial to test two hepcidin-guided screen-and-treat approaches against the standard-of-care 60 mg per day regimen. We calculated a threshold for the iron-regulatory hormone hepcidin that would indicate whether a participant was ready-and-safe to receive iron. Our study is, to the best of our knowledge, the first to assess hepcidin-guided antenatal iron supplementation. The weekly screen-and-treat approach was non-inferior to the standard-of-care regimen. No evidence was found that the screen-and-treat approach was safer with respect to adverse events or ex-vivo tests of *Plasmodium falciparum* growth in red blood cells or sentinel bacterial growth in plasma.

**Implications of all the available evidence**

We were unable to show any clear advantages of a hepcidin-guided screen-and-treat approach to maternal iron supplementation over current WHO recommendations. These data and other available evidence suggest that efforts should be directed towards developing low-cost iron supplements with better side-effect profiles to help overcome poor adherence that currently undermines antenatal iron-supplementation programmes.

We reasoned that hepcidin, the hepatic iron-regulatory peptide that acts as a master regulator of iron metabolism, could signal when women are ready-and-safe to receive iron and, hence, could form the basis of a screen-and-treat iron and folic acid supplementation regimen. Hepcidin is the homoeostatic regulator of body iron absorption, distribution, and metabolism.^16^ Circulating hepcidin is suppressed during iron deficiency, anaemia, and increased erythropoiesis, and amounts of hepcidin are increased by high levels of iron in serum and the liver and during infection and inflammation.^17^ By integrating these competing signals, a low amount of hepcidin indicates when the body is iron-deficient^18^, ^19^ and will efficiently absorb iron.^20^ Conversely, raised amounts of hepcidin would block duodenal iron absorption thereby rendering supplementation ineffective and exposing the gut microbiota to unnecessary iron that could cause dysbiosis and side-effects.^21^

We postulated that a hepcidin-guided screen-and-treat approach to iron supplementation would be non-inferior to WHO's recommended universal daily supplementation. Moreover, by lowering the total exposure to iron, we thought that this screen-and-treat approach might lead to better adherence and an improved side-effect and safety profile. Therefore, we designed the Hepcidin and Anaemia in Pregnancy (HAPn) study, a 12-week randomised, double-blind, non-inferiority trial in pregnant women from The Gambia, to assess these ideas.

## Methods

### Study design

Full details of the study design are in the appendix (pp 1, 2) and the published trial protocol.^22^ The HAPn study is a randomised, double-blind, proof-of-concept, non-inferiority trial to assess WHO's recommended daily iron regimen with two screen-and-treat approaches. We did the study in 19 rural communities in the Jarra West and Kiang East districts of The Gambia. In these locations, anaemia is common and malaria endemicity is low, heterogeneous, and seasonal.

Nurse midwives and fieldworkers identified and screened pregnant women at their first antenatal care visits (day 0) at two health facilities (Soma Health Centre, Soma Town, Jarra West; and Kaiaf Health Centre, Kaiaf Town, Kiang East), obtained informed consent, and gathered demographic information. Women aged 18–45 years were eligible for randomisation if gestational age was 14–22 weeks. Gestational age was assessed by either self-reported first date of last menstrual period or, if the woman could not recall this information, by fundal height. We excluded women if they were unlikely to remain in the area for the duration of the study, had severe anaemia (haemoglobin concentration <70 g/L), had a serious illness, had chronic disease, or self-reported a history of previous pregnancy complications (eg, repeated miscarriage or abortions, pre-eclampsia or eclampsia). At enrolment (day 0), women were provided with long-lasting insecticide-treated bed nets. Any woman found to have a concentration of haemoglobin lower than 70 g/L during the trial was treated as per the Gambian national protocol.

The trial was approved by the Medical Research Council (MRC) Unit The Gambia Scientific Coordinating Committee (SCC), Joint Gambia Government MRC ethics committee (SCC 1357, amendments L2014.56v2), and the London School of Hygiene & Tropical Medicine (LSHTM) ethics committee (no 7168). The trial was overseen by a data safety monitoring board, trial steering committee, and trial monitor, and it was done according to Good Clinical Practice standards supervised by the MRC Unit The Gambia at LSHTM (MRCG@LSHTM) Clinical Trials Office. All participants gave written informed consent.

### Randomisation and masking

At screening (day 0), eligible women were randomly allocated (1:1:1) using computer-generated numbers to one of three intervention arms: (1) WHO's recommended regimen of daily supplementation with UN University, UNICEF, and WHO international multiple micronutrient capsules (UNIMMAP) containing 60 mg iron as ferrous fumarate (the reference group); (2) weekly screening of plasma hepcidin for 12 weeks, every time succeeded by either daily supplementation for 7 days with UNIMMAP containing 60 mg iron if the concentration in plasma of hepcidin was less than 2·5 μg/L or daily supplementation for 7 days with UNIMMAP containing no iron if hepcidin levels were 2·5 μg/L or higher (the 60 mg screen-and-treat group); or (3) screen-and-treat supplementation as described for the 60 mg screen-and-treat group but with UNIMMAP containing 30 mg iron (the 30 mg screen-and-treat group). Calculation of the hepcidin threshold of less than 2·5 μg/L to define ready-and-safe to receive iron has been described previously.^19^ Randomisation was based on a permuted block design (block size of nine) with stratification by haemoglobin (above and below the median concentration of haemoglobin of the respective enrolment day) and gestational age (14–18 weeks or 19–22 weeks), to account for natural differences in haematological and iron status. Participants and the research team (except for the data manager) were unaware of group allocation and supplementation type throughout the fieldwork. Supplements were prepacked weekly by the field coordinator using computer-generated lists accounting for each participant's preceding hepcidin value. UNIMMAP was produced in three variants containing 60 mg, 30 mg, or no iron (DSM Nutritional Products, Johannesburg, South Africa) as identical gelatine capsules, packed in tubs under Good Manufacturing Practice conditions. All formulations also contained 400 μg folic acid and 13 other micronutrients (appendix p 3). Participants were instructed to take one capsule a day with water or another drink. The intervention started at day 0 (the day of screening, enrolment, and randomisation) and continued for 84 days or until delivery, whichever came first.

### Procedures

At screening (day 0), qualified personnel recorded the participant's medical history, did a medical examination, and collected a sample of venous blood (5–7 mL) for field measurement of haemoglobin (HemoCue Hb301 analyser; HemoCue, Ängelholm, Sweden) and to do the malaria rapid test (Alere Bioline Malaria Ag Pf, Abbot, Seoul, South Korea). If a blood sample was positive for *Plasmodium falciparum* infection on the malaria rapid test, we followed up with microscopy to confirm the presence of *P falciparum* parasites. Blood samples were transferred on ice to the laboratory at MRCG@LSHTM Keneba fieldstation for a full blood count (Medonic M Series; Boule Diagnostics, Spånga, Sweden) and assessment of plasma hepcidin. Plasma hepcidin was assayed by an ELISA with a detection range of 0·049–25·0 μg/L (hepcidin-25 [human] EIA Kit; Peninsula Laboratories International, San Carlos, CA, USA). The assay was validated as part of a worldwide harmonisation exercise.^23^ Hepcidin was quantified as single measurements to allow results within 24 h after blood collection and because of cost (appendix p 4). We also measured amounts in serum of ferritin, iron, unbound iron binding capacity, transferrin saturation, soluble transferrin receptor, C-reactive protein, and α_1_-acid glycoprotein, using an automated analyser (Cobas Integra 400 plus; Roche Diagnostics, Rotkreuz, Switzerland).

On day 2 and weekly thereafter, every participant was seen by a fieldworker who counted remaining supplements, measured axillary temperature, recorded self-reported side-effects, and gave the next week's supply of tablets. At day 14, day 49, and day 84, venous blood (5–7 mL) was gathered for assessments and processing, as described for day 0. At day 7 and weekly thereafter (except when venous blood was collected), field staff collected fingerprick capillary blood samples. At every timepoint, haemoglobin was measured by the HemoCue analyser, *P falciparum* infection was measured by the malaria rapid test, and hepcidin was assayed to ascertain subsequent allocation of iron or no iron in the two screen-and-treat groups. To maintain masking of the treatment allocation, participants in the reference group also had weekly fingerprick blood samples collected and hepcidin concentrations analysed, even though the results did not affect subsequent supplement allocation.

At day 0, day 14, day 49, and day 84, we used freshly washed red blood cells for malaria growth assays. Remaining plasma was stored at −20°C for iron and bacterial growth assays. Day 14 was selected for the ex-vivo malaria susceptibility assays as a time when there would most likely be a high level of reticulocytosis. Day 49 was then selected as the midpoint between days 14 and endpoint at day 84. Reticulocyte counts were assessed by fluorescence-activated cell sorting of CD71-positive cells.

Gambian national guidelines stipulate that pregnant women should receive intermittent preventive treatment against malaria with sulfadoxine and pyrimethamine, beginning with the first dose at 16 weeks of gestation and then at least two other doses with an interval of 1 month between them. To ensure no interference with the malaria susceptibility assays, we arranged for participants to receive their first dose of sulfadoxine and pyrimethamine immediately after the blood sample was taken on day 49.

We measured ex-vivo growth rates of *P falciparum* parasites in fresh red blood cells and of four sentinel bacterial species (*Staphylococcus epidermidis, Staphylococcus aureus, Salmonella enterica*, and *Escherichia coli*) in heat-inactivated serum as proxy safety indices, using methods described previously (appendix p 4).^15^, ^24^ The bacterial species were selected as frequent causes of sepsis in low-income settings and as representing a range of iron-acquisition mechanisms. Assays for *S epidermidis* proved unreliable, with frequent absence of any growth, so these findings have been excluded from the results. The technical reasons for this lack of growth were discovered in hindsight and insufficient samples were available to rerun the tests.

We monitored participants until delivery, and the outcomes of the pregnancy were registered for both mother and child (postnatal check-up within 72 h after delivery). When possible, reasons for a participant being lost to follow-up were recorded.

### Outcomes

The primary outcome was the amount of haemoglobin at day 84, measured as the difference in haemoglobin in each screen-and-treat group compared with the reference group (WHO's recommended regimen). Secondary outcomes were the prevalence at day 84 of anaemia, iron deficiency, and iron-deficiency anaemia, the total iron dose administered over the 84-day study period, adverse events, and adherence to the assigned strategy.^22^

Anaemia was defined as an amount of haemoglobin less than 11 g/dL. Iron deficiency was defined as a concentration in plasma of ferritin lower than 15 μg/L if C-reactive protein was lower than 5 mg/L, or plasma ferritin lower than 30 μg/L if C-reactive protein was higher than 5 mg/L. Iron-deficiency anaemia was defined as an amount of haemoglobin lower than 11 g/dL and plasma ferritin less than 15 μg/L when C-reactive protein was less than 5 mg/L, or haemoglobin lower than 11 g/dL and plasma ferritin less than 30 μg/L when C-reactive protein was higher than 5 mg/L and the ferritin index [soluble transferrin receptor:log_10_-ferritin] was greater than 2·0). Adverse events were defined as any untoward or unfavourable medical occurrence, including signs and symptoms associated temporally with the research procedure or trial intervention, whether considered related to the woman's participation in the research or not. Serious adverse events were investigated by a doctor and defined as any adverse event that was life-threatening or resulted in death or required admission to hospital or prolongation of admission, was a persistent or relevant disability or incapacity, was a congenital anomaly or birth defect, or was a reported maternal death, miscarriage, or stillbirth. Adherence was calculated as described in the appendix (p 5).

### Statistical analysis

For our sample size calculation, we used data from a previous study in neighbouring villages^25^ to analyse haemoglobin concentrations, which yielded an SD of 12·8 g/L. This value was used to calculate a sample size of 154 participants for each of the three arms, using a one-sided α of 2·5% with a conservative Bonferroni-type correction. Initially, a total sample size of 462 pregnant women was calculated, assuming less than 10% loss to follow-up. With a non-inferiority margin of −5·0 g/L, this number was used to provide 80% power to establish that the 60 mg screen-and-treat approach is non-inferior to WHO's recommended regimen, the 30 mg screen-and-treat approach is non-inferior to WHO's recommended regimen, and the 30 mg screen-and-treat approach is non-inferior to the 60 mg screen-and-treat approach. To ensure that the study was done across different seasons and to ensure that detailed monitoring could be achieved, we enrolled study participants in six cohorts starting from June, 2014, then roughly every 3–4 months afterwards, from September, 2014, January, 2015, April, 2015, August, 2015, and December, 2015. After the first two cohorts were enrolled, permission was obtained from the ethics committee to increase the sample size to 498, because loss to follow-up exceeded 10%.

Per-protocol analysis was used to assess non-inferiority of the primary outcome. All missing values and outliers present after data lock (on March 13, 2017) were maintained. In the intention-to-treat analysis, missing values were replaced by multiple imputation (appendix p 5). Intervention effects on continuous variables were measured as the difference in mean estimates, with logarithmic transformation (ln) as appropriate. A modified intention-to-treat analysis was also done (excluding participants withdrawn before the first dose of iron supplementation), and groups were compared using linear regression analysis, with intervention entered as a dummy-coded categorical variable. For analyses of bacterial growth, differences between timepoints were assessed by repeat measures ANOVA and Scheffé's post-hoc tests. Differences between study groups were assessed by χ^2^ test.

The number of adverse events was too low to allow meaningful analysis by type of adverse event. For every woman, we summed the counts for various types of adverse events. We used negative binomial regression to assess group differences in observed counts. Negative binomial regression was used instead of Poisson regression to account for overdispersion (ie, where the variance exceeds the mean). Effect sizes thus obtained are reported as the relative change in observed counts. Adherence was assessed as the extent to which the participant's history of supplementation coincided with the prescribed supplementation (appendix pp 5–7).

### Role of the funding source

The funder had no role in study design, data collection, data analysis, data interpretation, or writing of the report. The corresponding author had full access to all data in the study and had final responsibility for the decision to submit for publication.

## Results

**Figure 1:**
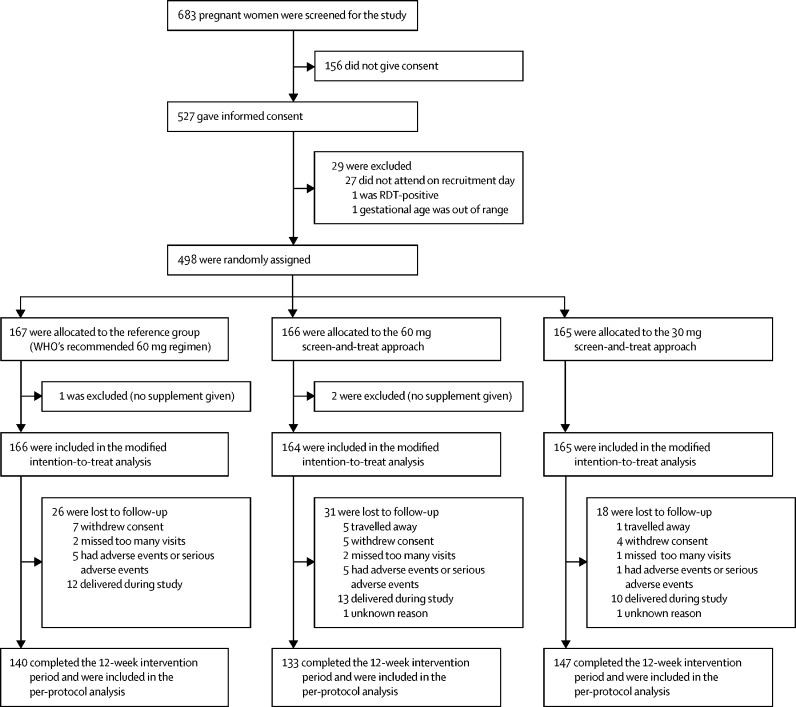
Trial profile RDT=rapid diagnostic test.

Between June 16, 2014, and March 3, 2016, we identified 683 pregnant women with a gestational age of 14–22 weeks, of whom 527 consented to take part in the trial. Of these pregnant women, 29 were excluded, mainly because they did not attend on recruitment day (figure 1). Six cohorts were enrolled, with 51 women enrolled from June, 2014, then 87 from September, 2014, 99 from January, 2015, 75 from April, 2015, 96 from August, 2015, and 90 from December, 2015. Of these 498 participants who were enrolled, 167 were allocated to WHO's recommended regimen (the reference group), 166 were allocated to the 60 mg screen-and-treat approach, and 165 were allocated to the 30 mg screen-and-treat approach. Among these 498 participants, 78 (16%) were withdrawn or lost to follow up before the scheduled completion of the intervention, with no evidence of a marked imbalance in non-completion between groups (figure 1). The per-protocol population therefore included 420 women, of whom 140 were assigned to WHO's recommended regimen, 133 were allocated to the 60 mg screen-and-treat approach, and 147 were allocated to the 30 mg screen-and-treat approach. Three participants were excluded before the first supplement was received, resulting in 495 women being included in the modified intention-to-treat analysis.

Participants' characteristics at enrolment were similar between study groups (appendix pp 8–11) and indicated a population with high prevalence of anaemia (>50%). A third of all women were iron-deficient using ferritin thresholds adjusted for inflammation. Poor iron status in the study population was confirmed by the high prevalence of other iron markers with abnormal values. About a third of participants had inflammation, as measured by C-reactive protein and α_1_-acid glycoprotein. Sickle-cell disorder was absent. Only one participant had a positive test for *P falciparum* infection.

**Figure 2:**
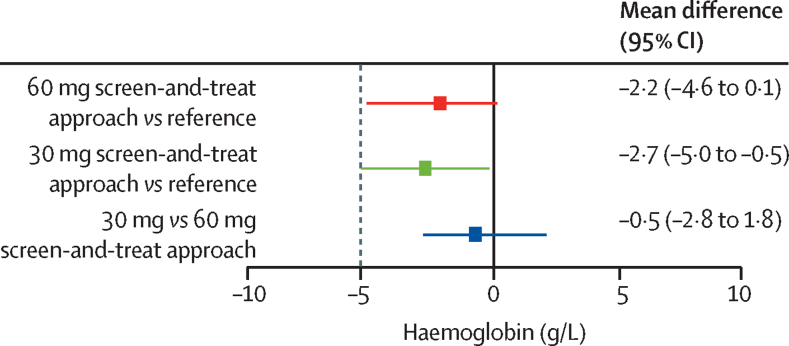
Non-inferiority tests Per-protocol analysis of change in haemoglobin from day 0 to day 84. Values are mean difference (95% CI). Dotted line shows the preset non-inferiority margin of −5 g/L.

In the per-protocol analysis, the difference in haemoglobin at day 84 did not exceed the preset non-inferiority margin of −5 g/L in the two screen-and-treat groups when compared with the reference group (table 1; figure 2). Moreover, the 30 mg screen-and-treat approach did not differ substantively from the 60 mg screen-and-treat approach (figure 2). Findings were similar in the modified intention-to-treat analysis (*vs* the reference group [n=166], −1·3 g/L, 95% CI −3·5 to 1·0 with the 60 mg screen-and-treat approach [n=164], and −2·9 g/L, −5·1 to −0·7 with the 30 mg screen-and-treat approach [n=165]).

**Table 1:** Primary and secondary outcomes, continuous variables

	**Participants with data available (n)**	**Mean estimate (SE or GSD)**	**Effect (95% CI)**
Haemoglobin at day 84 (g/L)*			
Reference group	139	110·1 (0·8)	··
60 mg screen-and-treat approach	131	107·9 (0·8)	–2·2 (–4·6 to 0·1)
30 mg screen-and-treat approach	145	107·4 (0·8)	–2·7 (–5·0 to −0·5)
**Hepcidin at day 84 (μg/L)**†‡			
Reference group	140	6·3 (3·4)	··
60 mg screen-and-treat approach	132	3·3 (4·2)	0·52 (0·37 to 0·75)
30 mg screen-and-treat approach	147	2·3 (4·7)	0·37 (0·26 to 0·52)
**Ferritin at day 84 (μg/L)**†			
Reference group	139	34·6 (1·9)	··
60 mg screen-and-treat approach	130	23·1 (1·8)	0·67 (0·58 to 0·77)
30 mg screen-and-treat approach	145	21·4 (1·7)	0·62 (0·54 to 0·71)
**Ferritin, inflammation-adjusted, at day 84 (μg/L)**†			
Reference group	139	31·6 (0·1)	··
60 mg screen-and-treat approach	130	21·2 (0·1)	0·67 (0·58 to 0·77)
30 mg screen-and-treat approach	145	19·3 (0·1)	0·61 (0·53 to 0·70)
**Ferritin index at day 84**†§			
Reference group	139	2·2 (1·5)	··
60 mg screen-and-treat approach	129	2·9 (1·5)	1·35 (1·23 to 1·49)
30 mg screen-and-treat approach	145	3·1 (1·5)	1·43 (1·31 to 1·58)

Logarithmic transformation (ln) was done for hepcidin and ferritin variables. GSD=geometric SD.

* Estimates are mean (SE); SE obtained by the Delta method. Effect is absolute difference in mean estimate versus the reference group.

† Estimates are geometric mean (GSD). Exponentiation of ln-transformed variables yielded effects expressed as ratios of geometric mean estimates versus the reference group.

‡ Estimates obtained using Tobit regression on ln-transformed hepcidin concentrations were left-censored at 0·049 μg/L (limit of detection) and right-censored at 25 μg/L.

§ Ferritin index is the ratio soluble transferrin receptor: log_10_-ferritin.

Compared with the reference group, hepcidin, ferritin, and the ferritin index (measures of iron deficiency) were all significantly lower at day 84 in both screen-and-treat groups (table 1). Values for other iron markers (eg, serum iron, transferrin, soluble transferrin receptor, and unbound iron binding capacity) confirmed these results (appendix pp 12–16). The prevalence of anaemia and iron deficiency showed a similar picture, although the contrasts between groups were more striking (table 2). In the reference group, the prevalence of anaemia dropped from 58% at day 0 to 45% at day 84 but rose in the two screen-and-treat groups, from 52% at day 0 to 57% at day 84 with the 60 mg screen-and-treat approach, and from 53% at day 0 to 59% at day 84 with the 30 mg screen-and-treat approach, such that the two screen-and-treat approaches were clearly inferior to WHO's recommended regimen. The prevalence of being ready-and-safe to receive iron declined substantially in the reference group, from 56% at day 0 to 21% at day 84, which was a lower prevalence at day 84 than in both screen-and-treat groups (42% in the 60 mg screen-and-treat group and 52% in the 30 mg screen-and-treat group), indicating better iron status in the reference group (appendix p 23). The prevalence of iron-deficiency anaemia showed a greater decline in the reference group (39% at day 0 to 17% at day 84) than in the 60 mg screen-and-treat group (from 40% to 29%), and in the 30 mg screen-and-treat group the prevalence increased slightly (from 37% to 40%). Iron deficiency prevalence defined using a soluble transferrin receptor threshold of greater than 4·4 mg/L showed a very similar pattern, with the prevalence of iron-deficiency anaemia also higher in both screen-and-treat groups at day 84 (appendix pp 17–19).

**Table 2:** Secondary outcomes, categorical variables

	**Number of participants/total with data available (n/N)**	**Prevalence (%)**	**Effect (95% CI)**
**Anaemia at day 84 (haemoglobin <110 g/L)**			
Reference group	63/139	45%	··
60 mg screen-and-treat approach	75/131	57%	11·9 (0·1 to 23·8)
30 mg screen-and-treat approach	86/145	59%	14·0 (2·5 to 25·5)
**Ready-and-safe to receive iron at day 84 (hepcidin <2·5 μg/L)**			
Reference group	30/140	21%	··
60 mg screen-and-treat approach	55/132	42%	20·2 (9·4 to 31·1)
30 mg screen-and-treat approach	77/147	52%	31·0 (20·4 to 41·5)
**Ferritin index >2·0 at day 84**			
Reference group	82/140	59%	··
60 mg screen-and-treat approach	116/133	87%	28·6 (18·7 to 38·6)
30 mg screen-and-treat approach	131/147	89%	30·5 (21·0 to 40·1)
**Iron-deficiency anaemia at day 84**			
Reference group	24/140	17%	··
60 mg screen-and-treat approach	38/131	29%	11·9 (1·9 to 21·8)
30 mg screen-and-treat approach	58/146	40%	22·6 (12·5 to 32·7)
**Iron dosage (% of weeks in which iron was received)**			
Reference group	1974/1974*	100%	··
60 mg screen-and-treat approach	1025/1905*	46%	–53·8 (–56·0 to −51·6)
30 mg screen-and-treat approach	952/2009*	53%	–47·4 (–49·6 to −45·2)
**Adherence**			
Reference group	275/1974*	86%	··
60 mg screen-and-treat approach	260/1905*	86%	0·3 (0·3 to 0·3)
30 mg screen-and-treat approach	246/2009*	86%	1·7 (1·7 to 1·7)

* Data are cases/person-weeks.

Adherence to daily supplementation was 86% in all study groups and was similar between groups (table 2). Participants in the 60 mg and 30 mg screen-and-treat groups received, respectively, 46% and 53% of the number of supplemental iron doses received by their peers in the reference group.

The frequency of adverse events and serious adverse events was similar between study groups (table 3). The frequency of self-reported illnesses and side-effects (ie, black stool, constipation, dizziness, fatigue, nausea, and stomachache) was similar in the 60 mg screen-and-treat group (14%) and in the reference group (11%; difference 2·5%, 95% CI 0·3 to 4·8); in the 30 mg screen-and-treat group the frequency was lower than in the reference group (8%; difference −3·5%, 95% CI −5·4 to −1·6). The most frequent side-effect was stomachache, which was similar in the 60 mg screen-and-treat group (82 cases per 1906 person-weeks) and in the reference group (81 cases per 1974 person-weeks; effect 1·0, 95% CI 0·7 to 1·6); in the 30 mg screen-and-treat group the frequency of stomachache was slightly lower than in the reference group (58 cases per 2009 person-weeks; effect 0·7, 95% CI 0·5 to 1·1; appendix pp 20–22). No participants died during the study.

**Table 3:** Safety outcomes

	**Cases/person-weeks**	**Observed number of events**	**Effect (95% CI)**
**Reported side-effects (aggregate score)**			
Reference group	220/1974	111 per 1000 person-weeks	··
60 mg screen-and-treat approach	261/1906	135 per 1000 person-weeks	1·2 (0·8 to 1·8)
30 mg screen-and-treat approach	154/2009	78 per 1000 person-weeks	0·7 (0·5 to 1·0)
**Adverse events**			
Reference group	167/1902	89 per 1000 person-weeks	··
60 mg screen-and-treat approach	149/1861	82 per 1000 person-weeks	–7·4 (–26·0 to 11·1)
30 mg screen-and-treat approach	175/1945	89 per 1000 person-weeks	1·6 (–17·2 to 20·3)
**Serious adverse events (DSMB notified)**			
Reference group	9/1904	29 per 10 000 person-weeks	··
60 mg screen-and-treat approach	14/1861	47 per 10 000 person-weeks	18·7 (–12·3 to 49·8)
30 mg screen-and-treat approach	6/1945	18 per 10 000 person-weeks	–10·2 (–34·0 to 13·6)

Individual complaints and adverse events are listed in the appendix (pp 20–22). Estimates of adverse events and serious adverse events were based on a negative binomial model, accounting for differences in exposure. The effect and 95% CI are the respective exponentiated relative changes in observed counts and their CIs. DSMB=data safety monitoring board.

**Figure 3:**
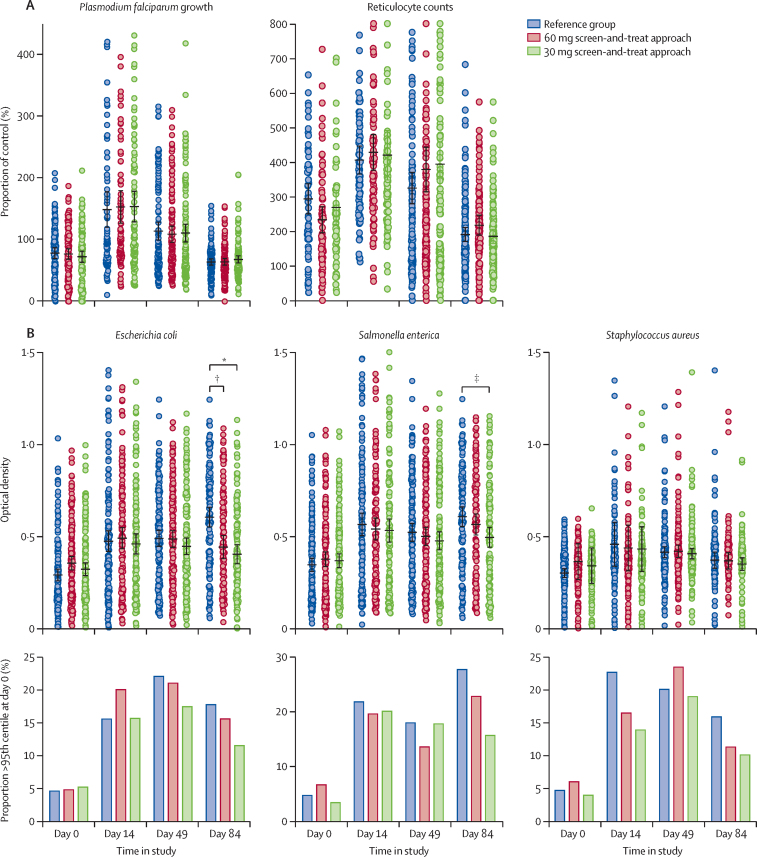
Ex-vivo assays of malaria growth in erythrocytes and sentinel bacteria growth in serum Left panel in (A) shows growth rates of *Plasmodium falciparum* strain FCR3-FMG in fresh RBCs relative to growth in RBCs from non-anaemic controls. Right panel in (A) shows reticulocyte counts relative to non-anaemic controls. Black lines show mean values and error bars show SEs. Compared with day 0, parasite growth and reticulocyte counts were significantly higher at day 14 (p=0·0012) and day 49 (p=0·0014), with no differences between treatment groups. Upper section in (B) shows individual participant data. Black lines show mean values and error bars show SEs. Compared with day 0, faster growth rates were seen on day 14, day 49, and day 84, for all species (p<0·0001 for all times). Lower plots in (B) show the proportion of serum samples from participants in which ex-vivo growth rates were greater than the 95% percentile, calculated on day 0 across all groups. All organisms showed significant increases after iron supplementation (p=0·0090). No differences between study groups were noted. RBC=red blood cell. *p<0·0001. †p<0·0001. ‡p=0·0011.

The growth of malaria parasites in fresh red blood cells was suppressed at day 0 (compared with non-anaemic controls), was greatly stimulated at day 14, and had gradually declined to day 0 levels by day 84, with no differences between study groups at any timepoint (figure 3A). Compared with day 0, before iron supplementation began, ex-vivo growth of *E coli, S enterica*, and *S aureus* was significantly faster at day 14, day 49, and day 84 (figure 3B). On day 14 and day 49, no differences were noted between study groups. On day 84, serum samples from women in the reference group supported faster bacterial growth of *E coli* and *S aureus* compared with women in the 30 mg screen-and-treat group, and *E coli* growth was faster at day 84 in the reference group compared with the 60 mg screen-and-treat group. This effect was attributable to the acute effect of iron administered to women in the 60 mg and 30 mg screen-and-treat groups, who had amounts of hepcidin measured 7 days previously below the 2·5 μg/L threshold. The appendix (p 24) shows no difference in bacterial growth across study groups among women who received iron 3 h before the blood draw and significantly lower growth in those in the 60 mg and 30 mg screen-and-treat groups who did not have iron.

## Discussion

The findings of our study show that iron exposure was reduced by half in the 60 mg screen-and-treat group (46% as much iron) and reduced by three-quarters in the 30 mg screen-and-treat group (27% as much iron). The screen-and-treat approaches were both non-inferior to WHO's recommended 60 mg daily regimen, with respect to the primary outcome of difference in amount of haemoglobin at day 84, according to our definition, with the lower limit of the confidence intervals falling within the preset non-inferiority margin. However, all secondary outcome measures of iron status showed evidence of inferiority. The prevalence of anaemia declined with the WHO recommended regimen but increased in both screen-and-treat groups. Likewise, the prevalence of iron deficiency and iron-deficiency anaemia was higher in both screen-and-treat groups compared with the reference group. Even the reference group had low apparent efficacy, with a 3·3 g/L improvement in haemoglobin over the 84-day study period and only a 13% reduction in anaemia, despite being implemented under the ideal conditions of an efficacy trial. However, true efficacy in ameliorating the haemodilution of pregnancy cannot be judged in the absence of a placebo arm.

Iron is a problematic nutrient with both beneficial and potentially harmful effects. Some of these effects can be serious, particularly in low-income settings where infections are common.^26^ Detection of differences in prevalence of serious infections would need a very large trial and, in the case of malaria, would be unethical because intermittent preventive treatment for pregnant women is advised, and in The Gambia it is mandated. We also issued insecticide-treated bed nets to all participants at enrolment. In view of these constraints, we used proxy assays of likely infection potential for malaria and for three sentinel bacteria that use a range of iron-acquisition mechanisms. By doing these ex-vivo assays at day 0, on day 14 and day 49 we were able to capture short-term and medium-term effects of chronic iron administration. On day 84, blood was drawn 3 h after the last oral iron (or non-iron) supplement and, hence, results at day 84 capture both chronic and acute post-absorptive effects of iron. Malaria parasite assays have previously provided a robust mechanism to account for how iron-deficiency anaemia protects against *P falciparum* infection (parasite invasion and growth rates are poor in older microcytic red blood cells) and why supplementation abrogates this effect (parasite invasion and growth rates are high in reticulocytes and large young red blood cells).^15^, ^27^, ^28^ These effects are replicated in our study and concur with associated changes in CD71 (a reticulocyte marker). No difference in plasmodial growth was noted between treatment groups at any timepoint, possibly because the most iron-deficient participants in all groups received iron early in the trial, which elicited a broadly similar reticulocyte surge despite the poorer overall performance of the two screen-and-treat groups. Reticulocytosis is also a natural response to the expansion of blood volume in mid-pregnancy and might have contributed to the increased risk of infections.^29^ Note that the absence of an acute effect of iron administration at day 84 is entirely consistent with the fact that the assay uses washed red blood cells and their susceptibility is governed by cell morphology rather than iron content.^24^

Growth rates of all three bacteria rose strikingly in all treatment groups after commencement of iron supplementation. In the absence of a placebo group, we cannot conclude that this increase is an effect of iron (or other micronutrients), but it seems highly likely. Pregnancy-related changes in humoral immunity are an unlikely explanation since plasma was heat-inactivated before inoculation. Furthermore, the growth-stimulatory effect of iron is clearly shown by the response to the acute iron and micronutrient administration 3 h before the blood draw on day 84. This finding corroborates our previous results in adult men, in whom bacterial growth rates were promoted by previous iron (without additional micronutrients) and were highly correlated with amounts in serum of iron and transferrin saturation.^24^ These ex-vivo assays might not equate to the situation in vivo but are highly suggestive that bloodstream bacteria would grow faster at higher concentrations of iron and transferrin saturation and would, therefore, have a greater chance of overcoming immune defences.

The real or perceived side-effects of taking oral iron supplements are less serious than the threat of a major infection, but they are important insofar as they lead to poor adherence to iron supplementation. The lesser performance of the two screen-and-treat groups in resolving iron deficiency and iron-deficiency anaemia might have been acceptable if evidence showed that they were safer or had fewer side-effects, as we initially postulated. In fact, the prevalence of self-reported illnesses and side-effects was highest in the 60 mg screen-and-treat group, possibly because women in the reference group adapted to iron supplementation better than when administration was intermittent (with on and off weeks). As might be expected, the prevalence of illnesses and side-effects was lowest in the 30 mg screen-and-treat group. Note that the unusually high adherence in this study could reflect the influence of sensitisation and fieldworker encouragement and the fact that participants were aware that adherence was being monitored.

There are several possibilities why the screen-and-treat approach did not work as we expected. First, it is possible that weekly screening does not adequately capture the short-term dynamics of hepcidin's responses to recent iron intake^30^, ^31^ or intercurrent infections and inflammation and that more frequent screening is needed. Even if this were the case, and a point-of-care test were available, it would be entirely impractical to screen more frequently. A second possibility is that our hepcidin threshold, calculated to diagnose iron deficiency,^19^ did not adequately differentiate iron absorbers from iron blockers (because the derivation did not include information on iron absorption). A higher threshold might have yielded more frequent dosing and a higher efficacy but would have been less effective at reducing the total number of women given iron, and it should not have been necessary because we already prioritised sensitivity over specificity in selecting the threshold (appendix pp 1, 2). A lower threshold would have reduced efficacy yet further. Our surmise is that the large bolus doses of highly absorbable ferrous fumarate override the physiological mechanism of hepcidin-induced iron blockade evolved to regulate duodenal iron absorption from foods and, hence, iron continued to be absorbed in the reference group even in the face of raised hepcidin.

Our study had several strengths and some weaknesses. Hepcidin is theoretically the ideal index of ready-and-safe to receive iron and it very effectively reduced the amount of iron administered in an area with high anaemia prevalence. The study had adequate statistical precision for the main outcomes, was done to Good Clinical Practice standards, and had high adherence and relatively few dropouts. One limitation is that the sample size was insufficient to capture potentially rare adverse events, and another is that the trial was conducted in an area with low malaria transmission, high use of insecticide-treated bed nets, and intermittent preventive treatment for pregnant women and, hence, could not assess to what extent the screen-and-treat approach reduced the risk of malaria. Our proxy safety outcomes for malarial and bacterial infections provide intuitively solid outcomes but might not reflect in-vivo susceptibility. Provision of iron with multiple other micronutrients can be viewed as both a strength and a weakness: a strength because other nutrient deficiencies that might limit the acquisition or utilisation of iron should be eradicated, and a weakness because of possible nutrient–nutrient interactions (eg, zinc in UNIMMAP might compete with iron for absorption). Note also that UNIMMAP capsules are not enteric-coated, which will not affect aggregate iron availability but may cause loss of other micronutrients. Because all participants received the same UNIMMAP, except for differences in iron content, this concern would not affect comparison between study groups.

Previous evidence shows that intermittent iron supplementation in pregnancy is somewhat less efficacious than daily supplementation,^4^, ^11^ and we conclude from this study that a hepcidin-guided screen-and-treat strategy does not overcome this limitation. Our results are likely to be generalisable at least to other populations in LMICs with high prevalence of anaemia and iron deficiency and in a low malaria setting. Future alternatives to universal oral iron supplementation in pregnancy might include use of parenteral iron formulations such as ferric carboxymaltose, which can deliver up to 1000 mg elemental iron over a 15-min infusion. This approach will need evidence of cost-effectiveness and safety in low-income settings, together with development of infrastructure, to overcome barriers to implementation. Therefore, we support continued application of the current WHO guidelines but urge development of novel iron formulations with much better side-effect profiles to encourage improved adherence. The findings of our malaria susceptibility assays underscore the importance of the WHO guideline that iron administration in malarious areas should ideally be implemented in conjunction with adequate measures to prevent, diagnose, and treat malaria.^32^
