# Supplementary material for: Hepcidin-guided screen-and-treat interventions against iron-deficiency anaemia in pregnancy: a randomised controlled trial in The Gambia
Source: Lancet Glob Health. 2019 Oct 10;7(11):e1564–74. doi: 10.1016/S2214-109X(19)30393-6 (PMC7109523; doi:10.1016/S2214-109X(19)30393-6)
Supplement: Supplementary appendix [file mmc1.pdf]

THE LANCET
Global Health

Supplementary appendix

This appendix formed part of the original submission and has been peer reviewed. We post it as supplied by the authors.

Supplement to: Bah A, Muhammad AK, Wegmuller R, et al. Hepcidin-guided screen-and-treat interventions against iron-deficiency anaemia in pregnancy: a randomised controlled trial in The Gambia. *Lancet Glob Health* 2019; **7:** e1564–74.

Supplementary methods

The full trial protocol has been previously published [1].

Schematic representation of trial design

Supplementary Figure 1

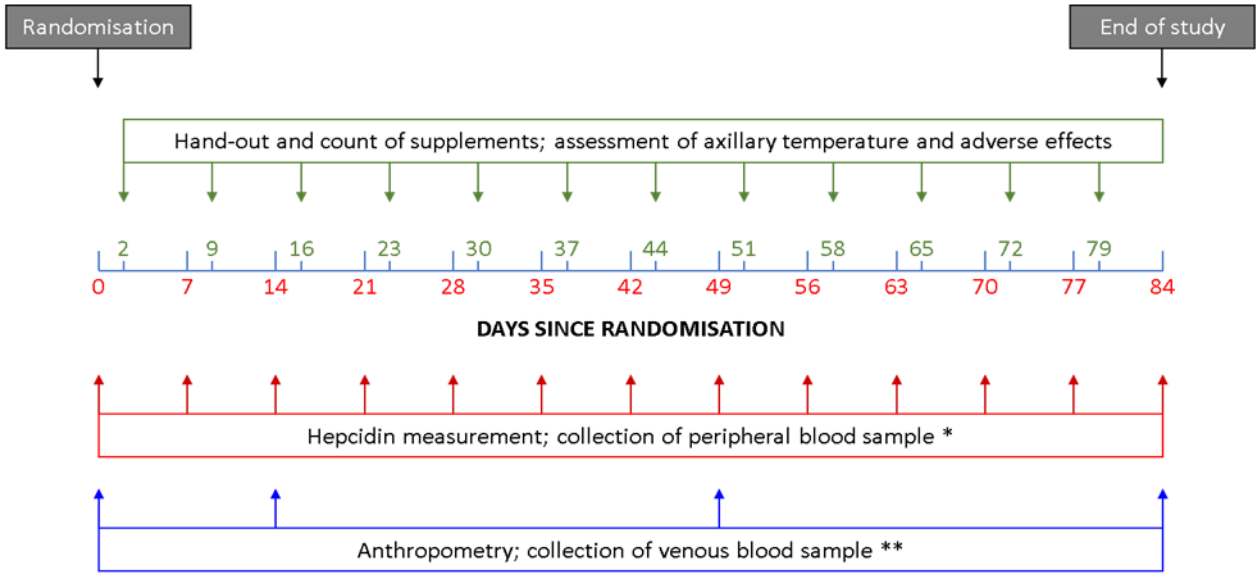

* For determination of haemoglobin concentration (HemoCue) and *P. falciparum* antigenaemia by rapid dipstick test

** For haemogram by automated blood analyser and to determine concentrations of iron markers, inflammatory markers, malaria and bacterial growth in plasma or serum

Derivation of the hepcidin threshold to define 'ready-and-safe' to receive iron

The hepcidin cut-off value of <2·5µg/L as a threshold to receive iron was based on the analysis of plasma from 395 pregnant women participating in the ENID study [2] with samples available for 3 time points (14wks, 20wks and 30wks gestation). Based on a reference standard of ferritin concentration <15µg/L and body iron content <0mg/kg, we constructed a receiver operating characteristic (ROC)-curve and calculated the area under the curve (AUC^{ROC}). The general suppression of hepcidin in pregnancy indicated that many women were ready to utilise iron. To make sure these women were not missed, we optimised for sensitivity over specificity, across the duration of pregnancy. At a hepcidin concentration of <2·5µg/L with a high Youden index, we had corresponding sensitivity and specificity values of 93·1% and 66·3% at 14wks, 86·1% and 52·2% at 20wks, and 86·2% and 84·9% at 30wks. The full method is described elsewhere [3].

.

Additional information on informed consent procedures

The Regional Health Team, local health staff and individual communities were informed and approved the study. We trained all field workers who took part in the recruitment of participants on translating the informed consent documents. We also translated the information sheet to all the illiterate participants in a language they understand in the presence of an independent witness. The literate participants read the information sheet in their own time. Participants were encouraged to ask questions and seek clarification from the field workers and the PI. We recorded by a signature or thumbprint the informed consent of all the participants who agreed to take part in the study.

Investigational product

The composition of the three formulations of UNIMMAP (containing 0, 30 and 60mg iron) is listed below.

Composition of the experimental supplement based upon the UNIMMAP formulation

Supplementary Table 1

Micronutrients	Dose/day	Ingredients
Vitamin A (µg RE)	800	Dry vitamin A acetate 325
Thiamine (mg)	1·4	Thiamine mononitrate
Riboflavin (mg)	1·4	Riboflavin
Niacin (mg)	18	Niacinamide
Vitamin B6 (mg)	1·9	Pyridoxine hydrochloride
Folic acid (µg)	400	Folic acid food grade
Vitamin B12 (µg)	2·6	Vitamin B12 0·1%
Vitamin C (mg)	70	Ascorbic acid
Vitamin D (IU)	200	Dry vitamin D3
Vitamin E (mg)	10	Dry vitamin E 50%
Zinc (mg)	15	Zinc oxide
Iron (mg)	60 or 30 or 0 (placebo)	Ferrous fumarate
Iodine (µg)	150	Potassium iodide 10% on Potato Maltodextrin
Selenium (µg)	65	Sodium selenite anhydrous
Copper (mg)	2	Copper gluconate

Additional details on blinding to intervention

Participants and the research team, with exception of the data manager, were blinded to the group allocation and supplementation type throughout the fieldwork. The supplements were pre-packed on a weekly basis by the field coordinator in Keneba using lists automatically computer generated by the data office taking into account the hepcidin results of the participants. The list indicated a participant's identity number, a letter and number (code W1 to W6) of the supplement type to be received by the participant in the subsequent 7 days, but the field coordinator did not know which code was allocated to which supplement or who belonged to which group.

Additional details on laboratory analyses

We measured hepcidin concentration in plasma from finger prick blood or from venous blood by competitive enzyme-linked immunosorbent assay (ELISA) (hepcidin-25 (human) EIA Kit, Bachem; now sold by Peninsula Laboratories International, San Carlos, USA) using a microplate photometer (Multiskan FC, Thermofisher Scientific, Waltham, MA, USA) with a detection range 0·049-25·0µg/L. Concentrations were interpolated from a 4-parameter curve fitted from a 2-fold, 10-point serial dilution made from a manufacturer-provided standard peptide. We quantified hepcidin as single measurements to allow results within 24 hours after blood collection and due to cost.

We prepared a haemogram from whole blood collected in EDTA tubes (Medonic M Series, Boule Diagnostics, Spånga, Sweden), and measured plasma concentrations of ferritin, iron, transferrin, soluble transferrin receptor (sTfR), C-reactive protein (CRP), and α_1-acid glycoprotein (AGP) using an automated analyser (Cobas Integra 400 plus, Roche Diagnostic, Rotkreuz, Switzerland). TSAT and UIBC were calculated.

We determined sickle cell status by performing haemoglobin electrophoresis in a Hu15 Standard Horizontal Gel Unit (Scie-Plas Ltd, Cambridge, UK) with a Shandon Vokam 400 power pack (Astmoor Rancorn, Cheshire, UK) in blood samples collected at baseline.

Bacterial growth assays

Staphylococcus aureus (strain NCTC8325), *Staphylococcus epidermidis* (FDA strain *PCI1200,* ATCC12228), *Salmonella enterica serovar* Typhimurium (strain *LT2*, ATCC19585) and *Escherichia coli* (strain *Crooks*, ATCC8739) were grown overnight for 18 hours at 37 °C in 5mL iron-free minimal growth media, Iscove's Modified Dulbecco's Medium (IMDM, Invitrogen). This was conducted in air with continuous shaking (250 rpm). All growth assays were run in triplicate in IMDM containing 50% heat-inactivated human serum. Bacterial growth was monitored by measuring the optical density at 620 nm (OD_{620}) hourly for 12 hours (*Staphylococcus aureus, Salmonella enterica serovar* Typhimurium, and *Escherichia coli)* and then at 20, 28, 36 hours (*Staphylococcus epidermidis*) using a Multiscan FC ELISA plate reader (Thermo Scientific).

Plasmodium growth assays

In vitro growth of the FCR3-FMG laboratory strain of *P falciparum* was assessed in fresh, washed RBCs as in[4] for 96h (performed in triplicate for RBCs from each study participant). RBCs from healthy, non-pregnant, adult iron replete donors of normal haemoglobin genotype and G6PD status not undergoing iron supplementation served as controls. Growth rates represent final 96h parasitaemia divided by initial 0h parasitaemia, analysed by flow cytometry[4].

Quantification of CD71-positive reticulocytes

CD71-positive reticulocytes in fresh RBCs were counted using PE-conjugated anti-human CD71 antibody (Clone M-A712, BD) and isotype control (Clone G155–178, BD), and analysed by flow cytometry for CD71-positive reticulocyte percentage relative to non-anaemic control as in[5].

Additional details on statistical analysis

Because plasma ferritin concentrations can be increased by inflammation independent of iron status, we adjusted for inflammation (concentrations of C-reactive protein and α_1-acid glycoprotein) measured in the same plasma samples, using approaches based on a Higher ferritin cutoff, and Excluding individuals. The higher ferritin cutoff means changing the cutoff to 30µg/L among those with inflammation, whilst the excluding individuals involves stratifying women into groups with and without inflammation and use ferritin values only among those without inflammation.

In the intention-to-treat analysis, missing values were replaced by multiple imputation using a Multiple Imputation Chained Equation with a burn-in of 100 and 100 imputations, including the following variables: gestational age, HemoCue haemoglobin concentration, hepcidin, red blood cells, mean corpuscular volume, red cell distribution width, haematocrit, mean platelet volume, white blood cells, Medonic haemoglobin, mean corpuscular haemoglobin concentration, lymphocytes, granulocytes, parity, gravida and age at start of study.

For individual women, we estimated adherence as the number of days that supplements were taken according to the capsule count (minus 2 days to account for the first two days after randomisation when supplementation was put on hold depending on the results of the first hepcidin concentration assessment) divided by the number of days between enrolment and leaving the study for reasons that were unrelated (or likely to be unrelated) to supplementation use (i.e., attaining the end of the 85-day intervention period, delivery, or emigration, whichever came first). Thus, as the denominator, we used the 85-day intervention period for women who refused, who were withdrawn for medical or unknown reasons, or who were withdrawn because of poor compliance. For groups, we calculated adherence by dividing the pooled number of days that supplements were taken as assessed by capsule count for all women by the pooled number of days until the end of the intervention period for all women. Details of calculation methods are shown in **Supplementary Figure 2.**

Methods used to describe supplement use in groups allocated to screen-and-treat supplementation with iron

Supplementary Figure 2

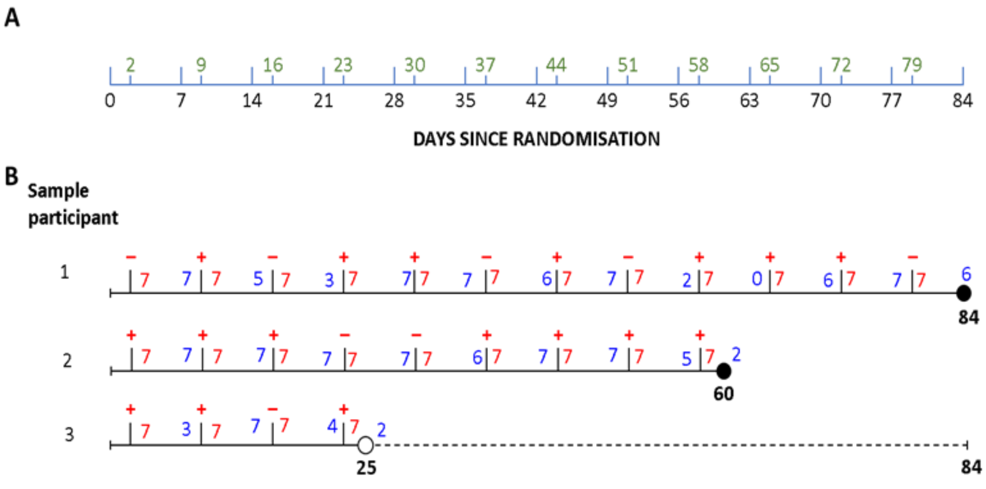

No. of supplements prescribed	No. of iron-containing supplements prescribed	No. of supplements consumed	No. of iron-containing supplements consumed
84	49	63	39
63	49	55	42
84	21	16	12

C

$$\text{Group adherence} = \frac{\text{Sum of number of supplements consumed by all participants}}{\text{Sum of number of supplements prescribed for all participants}}$$

$$\text{No. of supplements prescribed} = 84 + 63 + 84 = 231$$

$$\text{Percentage of prescribed supplements containing iron} = \frac{(49) + (49) + (21)}{(84) + (63) + (25)} = 69.2\%$$

LEGEND: Panel A: schematic representation of the scheduled intervention period and visits made to hand out supplements and to assess adherence. Panel B: follow-up time and exposure to supplementation for three individual study participants in the screen-and-treat groups (hypothetical data). Each participant was provided with 7 supplements per week for the duration of the study, but whether these supplements contained iron or placebo depended on hepcidin concentrations in plasma or serum samples collected two days earlier. Weekly measurement of hepcidin concentrations started at Day 0; weekly provision of supplements started at Day 2. Results of the plasma/serum tests are shown by +/– -signs, indicating hepcidin concentrations <2·5µg/L or ≥2·5µg/L, respectively. Values in red font indicate the number of iron-containing supplements provided each week; values of 0 indicate that participants were provided with placebo. Values in blue font indicate the number of supplements consumed in a single week (calculated as the number prescribed and handed out minus the number returned unconsumed), regardless of whether they contained iron or placebo. Values in bold font indicate the number of days that each participant took part in the study. Circles indicating the end of follow-up show whether the participant left the study for reasons considered unrelated to intervention (i.e., completed the 84-day intervention period, delivered, or migrated; closed circles), or for reasons that could be related to the use of supplementation (i.e. refusal, withdrawn for medical reasons, because of poor adherence, or for unknown reasons; open circles). In the latter case, the period between leaving the study and the scheduled end of intervention (dashed line) contributed to the follow-up time that was used as the denominator of the formula to calculate adherence. For example, participant 1 completed the intervention period of 84 days. In the first week of supplementation, which started at Day 2, she was provided with supplements containing placebo (hence, 0 iron-containing supplements), which she all consumed. Participant 3 refused further cooperation at Day 25; to calculate adherence, however, a follow-up period of 84 days was maintained. Panel C: Calculation of results (data from panel B).

All missing values and outliers present after the locking of the data were maintained and analysis performed with them for per protocol analysis. Since the data is longitudinal and at baseline most of the observations were non-missing, hence it would be ideal to use these values for the predictions of subsequent observations. Firstly, we set the data to identify the missing values, the imputation number (which would be zero at this point) and the multiple imputation identity. We start by registering the variables that would be required to impute the missing values. These variables included gestational age, HemoCue haemoglobin concentration, hepcidin, red blood cells, mean corpuscular volume, red cell distribution width, haematocrit, mean platelet volume, white blood cells, Medonic haemoglobin, mean corpuscular haemoglobin concentration, lymphocytes, granulocytes, parity, gravida and age at start of study. With these variables, we reshaped the data from long to wide, which simplified the data such that each individual now has only one row in the dataset. It also means that it is easy to use complete outcomes of some of the variables at some of the timepoints to predict the values of subsequent outcomes. We used a Multiple Imputation Chained Equation for the imputation with a burn-in of 100 and 100 imputations. This would also ensure that the Monte Carlo (MC) error is small enough to be unimportant[6]. The MC error was small enough to consider the number of imputations acceptable. The burn-in was adequate to show a convergence to a stationary state.

In the intention-to-treat analysis, missing values were replaced by multiple imputation. Intervention effects on continuous variables were measured as the difference in means, with logarithmic transformation as appropriate. We based the analysis of the primary end point (haemoglobin at Day 84) on the evaluation for non-inferiority with a per-protocol analysis. We also as per acceptable practice performed a modified intent-to-treat analysis (i.e., excluding participants who were lost to follow-up for being withdrawn before the first dose of supplement was received) on the randomised population and compared the groups using linear regression analysis, with intervention entered as a dummy-coded categorical variable and using the control arm (universal daily supplementation) as the reference group. To indicate non-inferiority, we used the lower limit of the 95% confidence interval for the difference in mean haemoglobin concentration between either of the screen-and-treat arms and the daily reference arm which should be above -5·0g/L (non-inferiority margin).

Supplementary results

Baseline characteristics of the subjects

See **Supplementary Table 2**. Groups were similar in baseline characteristics.

Baseline characteristics by intervention group

Supplementary Table 2:

**Characteristics**	**REFERENCE**		**S&T60**		**S&T30**	
	**n**	**Mean(SD)**	**n**	**Mean(SD)**	**n**	**Mean (SD)**
Age, years	166	27·1 (6·0)	164	27·1 (5·7)	165	27·1 (5·8)
Number of pregnancies*	166	3·0 [2·0, 5·0]	164	3·0 [2·0, 5·0]	165	4·0 [2·0, 5·0]
Number of previous live births*	166	2·0 [1·0, 4·0]	164	2·0 [1·0, 4·0]	165	3·0 [1·0, 4·0]
Gestation age, weeks	166	18·4 (2·5)	164	18·6 (2·6)	165	18·5 (2·7)
Height, cm	166	163·1 (6·5)	164	161·7 (6·1)	165	162·4 (6·4)
Weight, kg	166	59·5 (11·4)	164	59·9 (11·2)	165	59·1 (11·3)
Body mass index, kg/m^2	166	22·3 (3·8)	164	22·8 (3·7)	165	22·4 (4·0)
Sickle cell genotype (AS) ‡	33/162	20·4	24/162	14·8	29/162	17·9
Haemoglobin concentration, g/L						
By Medonic analyser	166	106·8 (13·7)	164	108·5 (14·2)	165	107·6 (14·5)
By HemoCue field photometer	166	112·0 (12·4)	164	113·9 (12·9)	165	113·1 (12·8)
Anaemia (haemoglobin <110g/L) ‡						
By Medonic analyser	96/165	58·2	85/163	52·2	87/165	52·8
By HemoCue field photometer	66/166	39·8	57/164	34·8	61/165	37·0
Haematocrit, %	166	29·3 (3·9)	163	29·8 (4·1)	164	29·4 (3·9)
Mean corpuscular volume (MCV), fL	166	79·6 (7·2)	163	79·1 (6·4)	164	78·8 (7·2)
MCV <85 fL‡	129/166	77·71	136/163	83·44	140/164	85·37

Mean corpuscular haemoglobin (MCH), pg	166	29·1 (3·1)	163	29·0 (2·7)	164	28·9 (3·1)
MCH <27 pg‡	31/166	18·7	32/163	19·6	31/164	18·9
Mean corpuscular haemoglobin concentration, (MCHC) g/dL	166	365·1 (11·2)	163	364·9 (10·8)	164	366·2 (10·7)
Erythrocyte distribution width, %*	166	13·3 [12·7, 14·4]	163	13·2 [12·6, 14·5]	164	13·2 [12·6, 14·8]
Leukocyte count, $\times 10^9$/L	166	7·5 (2·0)	163	7·5 (2·3)	164	7·2 (1·9)
Lymphocytes, $\times 10^9$/L†	166	1·9 (0·3)	163	1·9 (0·3)	164	1·8 (0·3)
Lymphocytes, %	166	27·7 (7·0)	163	27·8 (7·5)	164	27·1 (6·2)
Granulocytes, $\times 10^9$/L†	166	4·7 (0·4)	163	4·7 (0·4)	164	4·6 (0·3)
Granulocytes, %	166	65·6 (7·8)	163	65·4 (8·3)	164	66·2 (6·9)
Plasma marker concentrations						
Hepcidin, µg/L*	166	1·6 [0·4, 7·9]	164	2·5 [0·5, 8·4]	165	2·0 [0·5, 8·1]
Hepcidin <2·5 µg/L‡	93/166	56·0	83/164	50·6	92/165	55·8
Ferritin, µg/L*	161	21·2 [12·2, 40·3]	156	23·5 [11·5, 42·6]	152	22·4 [12·9, 42·6]
Iron deficiency (ferritin <15µg/L CRP <5 mg/L) OR (ferritin <30µg/L CRP >5 mg/L) ‡	64/166	38·6	65/164	39·6	61/165	37·0
Iron deficiency anaemia (Hb < 110 g/L ferritin < 15µg/L CRP <5mg/L) OR (Hb <110g/L ferritin < 30µg/L CRP >5mg/L & ferritin index > 2) ‡	42/165	25·5	46/162	28·4	36/163	22·1
Transferrin, g/L	162	3·3 (0·7)	160	3·3 (0·6)	160	3·3 (0·6)
Unsaturated iron binding capacity	164	55·0 (18·3)	160	55·5 (15·7)	163	56·2 (16·5)
(UIBC), µmol/L						
Iron, µmol/L	164	15·7 (8·6)	161	14·4 (7·0)	162	14·7 (7·0)
Iron <8·95 µmol/L‡	30/166	18·1	40/164	24·4	35/165	21·2
Transferrin saturation (TSAT) <16%‡	53/164	32·3	56/160	35·0	57/162	35·2

Soluble transferrin receptor, mg/L*	164	3·87 [2·85, 4·93]	160	3·99 [3·09, 5·10]	162	3·74 [2·93, 5·01]
Iron-deficient erythropoiesis (sTfR concentration > 4·4 mg/L) ‡	58/164	35·4	63/160	39·4	52/162	32·1
sTTfR log10Ferritin ratio (ferritin index)	154	2·78 (0·60)	149	2·91 (0·65)	149	2·83 (0·57)
Ferritin index >2‡	121/166	72·9	113/164	68·9	120/165	72·7
C-reactive protein (CRP), mg/L	163	5·1 (6·2)	161	7·1 (17·7)	161	5·6 (12·0)
α_1-acid glycoprotein (AGP), g/L	164	0·6 (0·2)	162	0·6 (0·3)	163	0·7 (0·3)
Inflammation‡						
CRP >5·0mg/L	52/163	31·9	45/161	28·0	45/161	28·0
AGP >1·0g/L	9/164	5·5	18/162	11·1	13/163	8·0
CRP >5·0mg/L OR AGP >1g/L	54/162	33·3	51/161	31·7	50/161	31·1
Current or recent *P falciparum* infection§	0/166	0·0	1/164	0·6	0/165	0·0

AGP: α_1-acid glycoprotein; CRP: C-reactive protein; sTfR: soluble transferrin receptor. Values indicate mean (SD), * median [IQR], or † geometric mean (SD), ‡ proportion in percentage, § As indicated by the presence in whole blood of histidine-rich protein II (HRP-II) antigen of *P falciparum*,

Full listing of all primary and secondary continuous variables at Day 84

See **Supplementary Table 3**. The HemoCue results were consistently about 5·0g/L higher than the Medonic results, but the relative differences between treatment arms were similar, though slightly more pronounced by HemoCue. In the S&T60 group the lower confidence interval for the difference against REF was close to the non-inferiority margin at Day 84 (-2·7g/L [-5·0g/L, -0·5g/L]). In the S&T30 group the lower confidence limit for the difference against REF was below the non-inferiority margin (-3·5g/L [-5·7g/L, -1·4g/L]).

Red cell counts (erythrocytes) were similar across the groups and the lower haemoglobin in the S&T groups was accounted for by lower mean corpuscular volume and Mean corpuscular haemoglobin as is consistent with their greater iron deficiency. With the exception of plasma iron in the S&T60 group, all measures of iron status were worse in the S&T groups than REFERENCE. There were no differences in markers of inflammation.

Trial outcome measures at Day 84 of intervention (per-protocol analysis), continuous variables

Supplementary Table 3:

Outcome	Intervention Group	n (%) of randomised	Estimate	SE¶	Effect [95% CI]
Haemoglobin concentration, g/L					
By Medonic analyser	REFERENCE	139 (83·7)	110·1	0·8	
	S&T60	131 (79·9)	107·9	0·8	-2·2 [-4·6, 0·1]
	S&T30	145 (87·9)	107·4	0·8	-2·7 [-5·0, -0·5]
By HemoCue field photometer	REFERENCE	140 (84·3)	116·6	0·8	
	S&T60	132 (80·5)	113·8	0·8	-2·7 [-5·0, -0·5]
	S&T30	147 (89·1)	113·0	0·8	-3·5 [-5·7, -1·4]
Haematocrit, %	REFERENCE	139 (83·7)	30·3	0·2	
	S&T60	131 (79·9)	29·8	0·2	-0·5 [-1·19, 0·13]
	S&T30	145 (87·9)	29·6	0·2	-0·7 [-1·3, -0·1]
Erythrocyte distribution width, % *	REFERENCE	139 (83·7)	13·55	0·01	
	S&T60	131 (79·9)	13·70	0·01	1·01 [0·98, 1·04]
	S&T30	145 (87·9)	13·67	0·01	1·01 [0·98, 1·04]
Erythrocyte count, $\times 10^{12}$/L	REFERENCE	139 (83·7)	3·7	0·0	
	S&T60	131 (79·9)	3·7	0·0	-0·0 [-0·1, 0·1]
	S&T30	145 (87·9)	3·7	0·0	-0·0 [-0·1, 0·1]
Mean corpuscular volume, fL	REFERENCE	139 (83·7)	82·53	0·5	

	S&T60	131 (79·9)	81·45	0·5	-1·08 [-2·6, 0·4]
	S&T30	145 (87·9)	81·14	0·5	-1·39 [-2·8, 0·1]
Mean corpuscular haemoglobin, pg	REFERENCE	139 (83·7)	30·0	0·2	
	S&T60	131 (79·9)	29·6	0·2	-0·5 [-1·1, 0·1]
	S&T30	145 (87·9)	29·5	0·2	-0·5 [-1·1, 0·1]
Mean corpuscular haemoglobin concentration, g/L	REFERENCE	139 (83·7)	363·9	0·9	
	S&T60	131 (79·9)	362·9	0·9	-1·1 [-3·6, 1·4]
	S&T30	145 (87·9)	363·4	0·9	-0·6 [-2·1, 1·9]
Leukocyte count, $\times 10^9$/L	REFERENCE	139 (83·7)	8·1	0·2	
	S&T60	791 (31·9)	7·9	0·2	-0·2 [-0·6, 0·3]
	S&T30	145 (87·9)	7·7	0·2	-0·5 [-0·9, -0·0]
Lymphocytes, $\times 10^9$/L †	REFERENCE	139 (83·7)	1·86	1·29	
	S&T60	131 (79·9)	1·88	1·30	1·01 [0·95, 1·07]
	S&T30	145 (87·9)	1·82	1·25	0·98 [0·93, 1·04]
Lymphocytes, %	REFERENCE	139 (83·7)	24·7	0·5	
	S&T60	131 (79·9)	25·4	0·5	0·7 [-0·7, 2·1]
	S&T30	145 (87·9)	25·5	0·5	0·7 [-0·6, 2·1]
Granulocytes, x10^9/L †	REFERENCE	139 (83·7)	5·38	1·34	
	S&T60	131 (79·9)	5·11	1·34	0·95 [0·89, 1·02]
	S&T30	145 (87·9)	5·00	1·37	0·93 [0·87, 1·00]
Plasma marker concentrations					

Hepcidin, µg/L *†	REFERENCE	140 (84·3)	6·26	3·44	
	S&T60	133 (81·1)	3·28	4·17	0·52 [0·37, 0·75]
	S&T30	147 (89·1)	2·32	4·74	0·37 [0·26, 0·52]
Ferritin, µg/L †	REFERENCE	139 (83·7)	34·56	1·87	
	S&T60	130 (79·3)	23·07	1·83	0·67 [0·58, 0·77]
	S&T30	145 (87·9)	21·42	1·74	0·62 [0·54, 0·71]
Transferrin, g/L	REFERENCE	139 (83·7)	3·1	0·1	
	S&T60	130 (79·3)	3·3	0·1	0·2 [0·0, 0·3]
	S&T30	146 (88·5)	3·4	0·1	0·3 [0·1, 0·4]
UIBC, µmol/L	REFERENCE	139 (83·7)	35·5	1·6	
	S&T60	13 (79·9)	42·6	1·6	7·1 [2·6, 11·6]
	S&T30	146 (88·5)	49·0	1·6	13·5 [9·1, 17·9]
Plasma iron, µmol/L	REFERENCE	140 (84·3)	32·7	1·3	
	S&T60	131 (79·9)	30·1	1·4	-2·7 [-6·3, 1·0]
	S&T30	146 (88·5)	25·2	1·3	-7·5 [-11·1, -3·9]
Soluble transferrin receptor, mg/L †	REFERENCE	140 (84·34)	3·25	1·39	
	S&T60	131 (79·88)	3·95	1·36	1·21 [1·13, 1·31]
	S&T30	146 (88·48)	4·03	1·36	1·24 [1·15, 1·33]
Ferritin index†	REFERENCE	139 (83·73)	2·15	1·53	
	S&T60	129 (78·66)	2·91	1·47	1·35 [1·23, 1·49]
	S&T30	145 (87·88)	3·08	1·48	1·43 [1·31, 1·58]

C-reactive protein, mg/L	REFERENCE	137 (82·5)	4·5	0·5	
	S&T60	129 (78·7)	4·2	0·5	-0·3 [-1·7, 1·0]
	S&T30	145 (87·9)	5·2	0·5	0·8 [-0·6, 2·1]
α1-acid glycoprotein, g/L	REFERENCE	140 (84·3)	0·5	0·0	
	S&T60	131 (79·9)	0·4	0·0	-0·0 [-0·1, 0·0]
	S&T30	146 (88·5)	0·48	0·0	0·0 [-0·0, 0·1]

* Estimates obtained using Tobit regression on the natural-log transformed values, such that at corresponding transformed values, erythrocyte distribution width was left-censored at 11·5% and right-censored at 25%, and hepcidin concentration was left-censored at 0·049µg/L (limit of detection) and right-censored at 25µg/L with results exponentiated and presented in the table.

Values indicate mean (SE) or † geometric mean (GSD as geometric standard deviation). Exponentiation of log-transformed variables † yielded effect estimates that are expressed as relative differences between geometric means.

¶ SE = standard error obtained by the Delta method.

Trial outcome measures at Day 84 of intervention (per-protocol analysis), categorical variables

Supplementary Table 4:

Outcome	Intervention Group	Prevalence, %	n/N	Effect [95% CI]
Anaemia (haemoglobin concentration <110g/L)				
By Medonic analyser	REFERENCE	45·3	63/139	Reference
	S&T60	57·3	75/131	11·93 [0·09, 23·77]
	S&T30	59·3	86/145	13·99 [2·48, 25·49]
By HemoCue field photometer	REFERENCE	26·4	37/140	Reference
	S&T60	28·0	37/132	1·60 [-8·98, 12·19]
	S&T30	32·0	47/147	5·54 [-4·95, 16·04]
Hepcidin concentration <2·5µg/L	REFERENCE	21·4	30/140	Reference
	S&T60	42·1	56/133	20·68 [9·88, 31·48]
	S&T30	52·4	77/147	30·95 [20·40, 41·51]
Mean corpuscular volume <85fL	REFERENCE	62·6	87/139	Reference
	S&T60	71·8	94/131	9·17 [-1·98, 20·31]
	S&T30	74·5	108/145	11·89 [1·17, 22·62]
Mean corpuscular haemoglobin <27pg	REFERENCE	7·2	10/139	Reference
	S&T60	10·7	14/131	3·49 [-3·32, 10·31]
	S&T30	12·4	18/145	5·22 [-1·65, 12·09]
Plasma ferritin concentration <15µg/L (not adjusted for inflammation	REFERENCE	8·6	12/140	Reference
	S&T60	21·1	28/133	12·48 [4·14, 20·82]

	S&T30	21·8	32/147	13·20 [5·07, 21·32]
Iron deficiency (ferritin <15µg/L CRP <5mg/L) OR (ferritin <30µg/L CRP >5mg/L)	REFERENCE	19·3	27/140	Reference
	S&T60	30·5	40/131	11·2 [1·0, 21·5]
	S&T30	41·1	60/146	21·8 [11·5, 32·1]
Iiron deficiency anaemia (Hb <110g/L ferritin < 15µg/L CRP <5mg/L) OR (Hb <110g/L ferritin < 30µg/L CRP >5mg/L & ferritin index >2)	REFERENCE	58·6	82/140	Reference
	S&T60	87·2	116/133	28·6 [18·7, 38·6]
	S&T30	89·1	131/147	30·5 [21·0, 40·1]
Plasma iron concentration <8·95µmol/L	REFERENCE	3·6	5/140	Reference
	S&T60	3·0	4/133	-0·56 [-4·79, 3·66]
	S&T30	6·8	10/147	3·23 [-1·87, 8·33]
Transferrin saturation (TSAT) <16%	REFERENCE	5·0	7/139	Reference
	S&T60	15·3	20/131	10·23 [3·08, 17·38]
	S&T30	19·9	29/146	14·83 [7·40, 22·25]
Iron-deficient erythropoiesis (sTfR concentration >4·4mg/L)	REFERENCE	14·3	20/140	Reference
	S&T60	38·2	50/131	23·88 [13·74, 34·02]
	S&T30	41·8	61/146	27·50 [17·62, 37·37]
Inflammation, CRP ≥5·0mg/L or AGP >1·0g/L	REFERENCE	33·6	46/137	Reference
	S&T60	23·3	30/129	-10·32 [-21·08, 0·43]
	S&T30	32·4	47/145	-1·16 [-12·14, 9·82]
Plasma ferritin <15µg/L and AGP <1·0g/L	REFERENCE	8·7	12/138	Reference

	S&T60	21·1	28/133	12·36 [3·98, 20·73]
	S&T30	22·1	32/145	13·37 [5·15, 21·60]
Plasma ferritin <15µg/L and CRP <5·0mg/L or AGP <1·0g/L	REFERENCE	10·6	10/94	Reference
	S&T60	21·4	22/103	10·72 [0·65, 20·80]
	S&T30	25·0	25/100	14·36 [3·83, 24·89]
Current or recent *P. falciparum* infection‡	REFERENCE	1·4	2/140	Reference
	S&T60	2·3	3/133	0·83 [-2·37, 4·03]
	S&T30	3·4	5/147	1·97 [-1·56, 5·50]

‡ As indicated by the presence in whole blood of histidine-rich protein II (HRP-II) antigen of *P. falciparum*

Reported side effects, adverse and serious adverse events

Supplementary table 5:

**Outcome**	**Intervention group**	**Observed counts per 1000 person-weeks**	**Cases/Person-weeks**	**Effect [95% CI]**
**Reported side effects**				
Nausea	REFERENCE	11	21/1974	
	S&T60	14	27/1906	1·4 [0·64, 2·86]
	S&T30	7	14/2009	0·7 [0·29, 1·51]
Dizziness	REFERENCE	31	62/1974	
	S&T60	40	76/1906	1·3 [0·8, 2·0]
	S&T30	16	32/2009	0·5 [0·3, 0·9]
Constipation	REFERENCE	13	26/1974	
	S&T60	22	42/1906	1·7 [0·8, 3·4]
	S&T30	14	28/2009	1·1 [0·5, 2·3]
Black stool	REFERENCE	5	9/1974	
	S&T60	2	3/1906	0·3 [0·1, 1·4]
	S&T30	3	6/2009	0·7 [0·2, 2·0]
Stomach ache	REFERENCE	41	81/1974	
	S&T60	43	82/1906	1·0 [0·7, 1·6]
	S&T30	29	58/2009	0·7 [0·5, 1·1]
Fatigue	REFERENCE	11	21/1974	
	S&T60	16	31/1906	1·5 [0·7, 3·2]
	S&T30	8	16/2009	0·8 [0·3, 1·8]
**Adverse events**				
Cough, cold and chest pain	REFERENCE	11	20/1902	
	S&T60	10	19/1861	1·0 [0·5, 1·8]
	S&T30	9	18/1945	0·9 [0·5, 1·7]
Diarrhoea	REFERENCE	3	5/1902	
	S&T60	2	3/1861	0·6 [0·1, 2·6]
	S&T30	2	4/1945	0·8 [0·2, 2·9]

Fever	REFERENCE	3	5/1902	
	S&T60	2	4/1861	0·8 [0·2, 3·0]
	S&T30	5	9/1945	1·8 [0·6, 5·3]
General body pain #	REFERENCE	4	7/1902	
	S&T60	3	5/1861	0·7 [0·2, 2·5]
	S&T30	4	8/1945	1·1 [0·4, 3·4]
Headache #	REFERENCE	11	21/1902	
	S&T60	9	17/1861	0·8 [0·4, 1·6]
	S&T30	12	24/1945	1·1 [0·6, 2·1]
Heartburn #	REFERENCE	2	3/1902	
	S&T60	2	3/1861	1·0 [0·2, 5·7]
	S&T30	6	11/1945	3·6 [0·9, 14·8]
Lower abdominal pain #	REFERENCE	20	38/1904	
	S&T60	16	30/1861	0·8 [0·5, 1·3]
	S&T30	15	30/1945	0·8 [0·5, 1·3]
Toothache	REFERENCE	6	11/1902	
	S&T60	2	4/1861	0·4 [0·1, 1·2]
	S&T30	3	6/1945	0·5 [0·2, 1·4]
Urinary tract infection or dysuria	REFERENCE	9	17/1902	
	S&T60	10	18/1861	1·1 [0·6, 2·1]
	S&T30	9	17/1945	1·0 [0·5, 1·9]
Vomiting	REFERENCE	1	2/1902	
	S&T60	2	3/1861	1·5 [0·3, 9·2]
	S&T30	1	2/1945	1·0 [0·1, 6·9]
Gastritis #	REFERENCE	2	3/1902	
	S&T60	4	7/1861	2·4 [0·6, 9·8]
	S&T30	2	4/1945	1·3 [0·3, 6·2]
Nausea	REFERENCE	1	1/1902	
	S&T60	1	1/1861	1·0 [0·1, 16·4]
	S&T30	1	2/1945	2·0 [0·2, 21·6]

Others	REFERENCE	18	34/1902	
	S&T60	19	35/1861	1·1 [0·7, 1·7]
	S&T30	20	40/1945	1·2 [0·7, 1·8]
**Serious adverse events¤**				
Death	REFERENCE	0	NA	
	S&T60	0	NA	NA
	S&T30	0	NA	NA
Life-threatening	REFERENCE	0	NA	
	S&T60	0	NA	NA
	S&T30	0	NA	NA
Hospitalisation Required or Prolonged	REFERENCE	3	1/1904	
	S&T60	10	3/1861	1·5 [0·2, 12·3]
	S&T30	0	0/3255	0·0 [0·0]
Congenital anomaly/birth defect	REFERENCE	0	NA	
	S&T60	0	NA	NA
	S&T30	0	NA	NA
Miscarriage	REFERENCE	20	4/1904	
	S&T60	31	5/1861	1·5 [0·2, 12·3]
	S&T30	4	1/1945	0·2 [0·0, 2·5]
Stillbirth #	REFERENCE	13	4/1904	
	S&T60	20	6/1861	1·6 [0·4, 5·6]
	S&T30	15	5/1945	1·2 [0·3, 4·5]

used Negative Binomial regression, otherwise Poisson regression used

¤ Calculated using: observed counts per 10,000 person weeks

Variation in plasma hepcidin through the course of the intervention and change over time of hepcidin and haemoglobin concentration, by intervention group (per protocol analysis)

Supplementary Figure 3

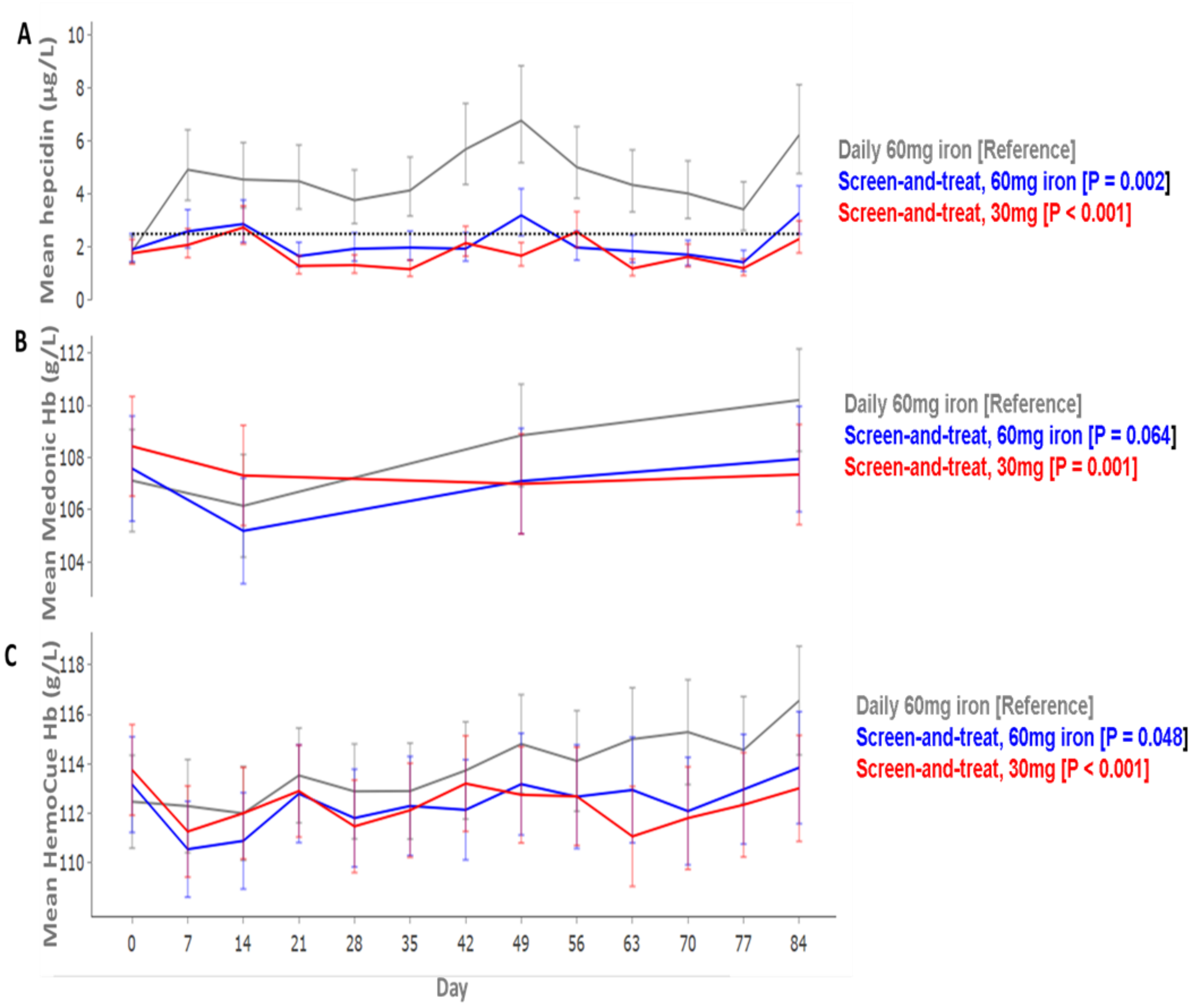

Legend:

Hepcidin concentration (**A**) was analysed by Tobit regression to account for right-censored values (see text). Mean values and 95%CI. P-values for time × intervention interaction effects: S&T60 versus REF, P = 0·002 and S&T30 versus REF, P < 0·001. Haemoglobin concentration measured by Medonic analyser (**B**): S&T60 versus REF, P = 0·064 and S&T30 versus REF, P = 0·001. Haemoglobin concentration measured by HemoCue photometer (**C**): S&T60 versus REF, P = 0·048 and S&T30 versus REF, P < 0·001.

Sentinel bacterial growth rates at Day 84 according to whether pregnant women received iron or placebo 3 h prior to blood draw

Supplementary Figure 4

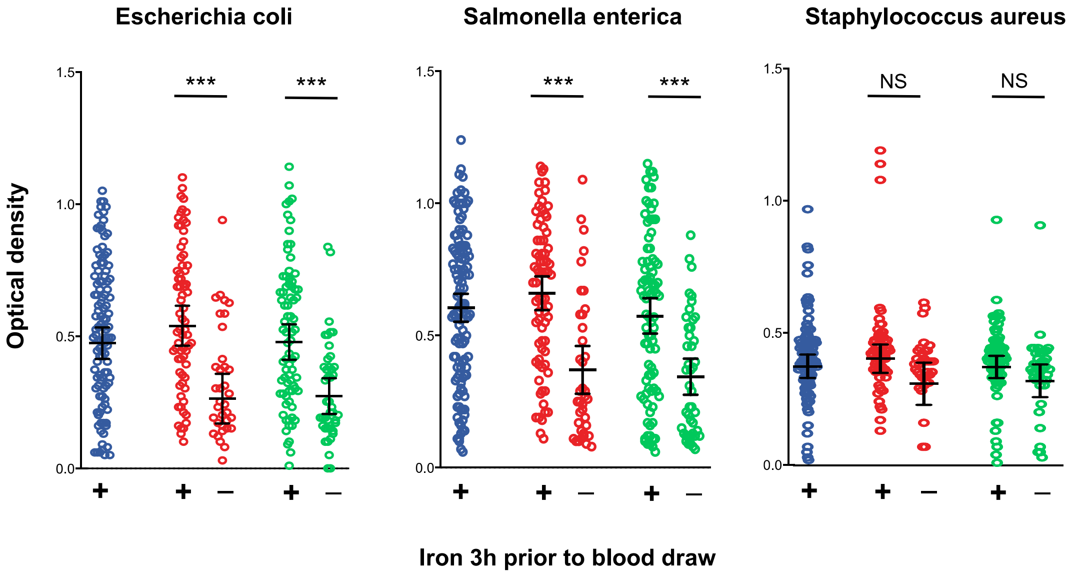

Legend:

Ex vivo bacterial growth rates in serum from Day 84. Blue = REF, red = S&T60, green = S&T30. On this final day of the study pregnant women received multiple micronutrients with iron (+) or without iron (-) according to their hepcidin level measured 7 days previously except in REF who all received multiple micronutrients with iron as per the protocol. *** = P<0·001, NS = not significant. In the women receiving iron there was no difference in mean growth between the three intervention groups.

References

1. Bah A, Wegmuller R, Cerami C, Kendall L, Pasricha SR, Moore SE, Prentice AM. A double blind randomised controlled trial comparing standard dose of iron supplementation for pregnant women with two screen-and-treat approaches using hepcidin as a biomarker for ready and safe to receive iron. BMC Pregnancy Childbirth. **16**; 157 (2016).

2. Moore SE, Fulford AJ, Darboe MK, Jobarteh ML, Jarjou LM and Prentice AM. A randomized trial to investigate the effects of pre-natal and infant nutritional supplementation on infant immune development in rural Gambia: the ENID trial: Early Nutrition and Immune Development. BMC Pregnancy Childbirth, **12**: 107 (2012).

3. Bah A, Pasricha SR, Jallow MW, Sise EA, Wegmuller R, Armitage AE, Drakesmith H, Moore SE, Prentice AM. Serum hepcidin concentrations decline during pregnancy and may identify iron deficiency: Analysis of a longitudinal pregnancy cohort in The Gambia. J Nutr. **147**; 1131-37 (2017).

4. Clark MA, et al. Host iron status and iron supplementation mediate susceptibility to erythrocytic stage Plasmodium falciparum. Nat. Commun. **5**; 4446 (2014).

5. Goheen MM, et al. Anaemia offers stronger protection than sickle cell trait against the erythrocytic stage of Falciparum malaria and this protection is reversed by iron supplementation. EBioMedicine. **14**; 123–130 (2016).

6. White IR, Royston P and Wood AM, *Multiple imputation using chained equations: Issues and guidance for practice.* Stat Med, 2011. **30**(4): p. 37
